# Reirradiation for local recurrence of oral, pharyngeal, and laryngeal cancers: a multi-institutional study

**DOI:** 10.1038/s41598-023-29459-2

**Published:** 2023-02-21

**Authors:** Hideya Yamazaki, Gen Suzuki, Norihiro Aibe, Hiroya Shiomi, Ryoong-jin Oh, Ken Yoshida, Satoaki Nakamura, Koji Konishi, Mikio Ogita

**Affiliations:** 1 Department of Radiology, Graduate School of Medical Science, Kyoto Prefectural University of Medicine, 465 Kajiicho Kawaramachi Hirokoji, Kamigyo-Ku, Kyoto, 602-8566 Japan; 2 CyberKnife Center, Soseikai General Hospital, Kyoto, 612-8473 Japan; 3 Department of Radiation Oncology, Miyakojima IGRT Clinic, Osaka, 534-0021 Japan; 4 Department of Radiology, Kansai Medical University, Hirakata, 573-1010 Japan; 5 Department of Radiation Oncology, Osaka International Cancer Institute, Osaka, Japan; 6 Radiotherapy Department, Fujimoto Hayasuzu Hospital, Miyakonojo, 885-0055 Japan

**Keywords:** Medical research, Oncology, Cancer

## Abstract

This study aimed to examine the efficacy and toxicity of reirradiation in patients with locally recurrent oral, pharyngeal, and laryngeal cancers. We conducted a retrospective, multi-institutional analysis of 129 patients with previously irradiated cancer. The most frequent primary sites were the nasopharynx (43.4%), oral cavity (24.8%), and oropharynx (18.6%). With a median follow-up duration of 10.6 months, the median overall survival was 14.4 months and the 2-year overall survival rate was 40.6%. For each primary site, the 2-year overall survival rates were 32.1%, 34.6%, 30%, 60.8%, and 5.7% for the hypopharynx, oral cavity, larynx, nasopharynx, and oropharynx, respectively. Prognostic factors for overall survival were primary site (nasopharynx versus other sites) and gross tumor volume (GTV) (≤ 25 cm^3^ versus > 25 cm^3^). The 2-year local control rate was 41.2%. Twenty-four patients (18.6%) presented with grade ≥ 3 toxicities, including nine with hemorrhages that led to grade 5 toxicities in seven patients. All nine tumors that caused hemorrhage showed tumor encasement of the carotid ≥ 180 degrees and eight of nine tumors had larger GTV > 25 cm^3^. Reirradiation is a feasible treatment option for small local recurrence of oral, pharyngeal, and laryngeal cancers, with the requirement of a strict eligibility assessment for large tumors with carotid encasement.

## Introduction

The main obstacle to the treatment of head and neck cancer after curative treatment, surgery, or radiotherapy with or without systemic therapy is a locoregional failure, occurring in 20–30% of patients^1–3^. Salvage treatment represented a clinical challenge due to frequently unresectable and/or locally advanced diseases. In the absence of curative salvage surgery, systemic therapy was the treatment of choice, and led to a short survival duration, with a median of 6–10 months^4,5^. Recent advanced radiotherapy techniques, including stereotactic body irradiation (SBRT) and in-tensity-modulated radiotherapy (IMRT), enable more precise treatment of recurrent cancers and help improve head and neck cancer outcomes^6–12^. Many studies have reported experiences of reirradiation, including ours; however, almost all studies had heterogeneity at primary sites (except, nasopharynx), recurrent sites (local or lymph nodes), and histology^6–12^. To provide detailed and essential information, we have attempted to evaluate the outcomes of reirradiation by focusing on the local recurrence of oral, pharyngeal, and laryngeal cancers, with or without lymph node involvement, using multi-institutional data.

Due to complex patient anatomy, and proximity of critical organs, curative treatment could be highly toxic to patients with a rate of grade 3 or higher acute toxicity as high as 80% and severe late toxicity rates range from 15 to 35% even in the initial radiotherapy^1–15^. As reirradiation has a potential risk to elevate toxicity than initial radiotherapy for cumulative high radiation dose, we should pay meticulous attention, especially for late severe toxicities such as mucositis, visual dis-order, trismus, fistula, infection/abscess, soft tissue, and/or bone necrosis. Additionally, we have examined the frequency and characteristics of severe hemorrhagic toxicities (i.e. carotid blowout syndrome [CBOS]), which were identified as one of the most serious consequences of reirradiation^7,8,13–15^. Therefore, this study aimed to examine the efficacy and toxicity of reirradiation using multi-institutional data focusing on the local recurrence of oral, pharyngeal, and laryngeal primary cancers.

## Materials and methods

### Patients

We included patients with locally recurrent oral, pharyngeal, and laryngeal cancers without metastasis treated at six institutions between 2000 and 2016 (CyberKnife Center, Soseikai General Hospital; Department of Radiation Oncology, Miyakojima IGRT Clinic; Radiotherapy Department, Fujimoto Hayasuzu Hospital, Department of Radiation Oncology, Osaka International Cancer Institute, Graduate School of Medical Science, Kyoto Prefectural University of Medicine) were recruited. All recurrences occurred in an area previously irradiated with ≥ 30 Gy dose in 10 fractions (equivalent 2-Gy fractions = EQD2 ≥ 36 Gy, using α/β = 3) and were diagnosed pathologically and/or radiologically. We included patients (i) recurrence after curative intent treatment, including chemotherapy or surgery, with radiotherapy. (ii) Eastern Cooperative Oncology Group performance status of 0–2. (iii) inoperable status according to the opinion of head and neck surgeon (information not only surgical treatment but also systemic therapy depended on patient status; performance status, comorbidity, and patient’s will).

We excluded patients with lymph node metastasis without local failure, and with distant metastasis. We collected demographics (age and sex), tumor factor (site, primary or primary plus lymph node metastasis, size, and volume), treatment (previous surgical history, concurrent chemotherapy, initial radiotherapy, dose fractionation and procedure, duration from the previous radiotherapy to reirradiation, reirradiation dose, fraction, and treatment procedure), and outcome (overall survival, local tumor control, and lymph node metastasis, distant metastasis, acute and late toxicity). The first course of radiotherapy was administered with either curative intent or postoperative radiotherapy for histologically proven malignancy (squamous cell carcinoma). All patients had an Eastern Cooperative Oncology Group performance status of 0–2. A total of 118 patients underwent stereotactic radiotherapy (CyberKnife; Accuray Inc, C.A. United States), and 11 underwent IMRT. Visible tumors in imaging studies were defined as the gross tumor volume (GTV) and expanded to planning target volume (PTV) with an additional adequate margin. Patients were treated with a median dose of 30 Gy (range, 20–60 Gy) in a median of five fractions (range, 5–20 fractions). The most frequently administered doses were 30 Gy (n = 30), 35 Gy (n = 20), 27 Gy (n = 12), and 32 Gy (n = 10). The biologically equivalent dose was calculated as EQD2 using a linear quadratic model and was estimated according to the following equation: EQD2 = n × d × ([α/β) + d]/[α/β) + 2]), where n was the number of treatment fractions, d was the dose per fraction in Gy, α/β = 10 for tumors, and α/β = 3 for organs at risk. We divided EQD2 by a median ≤ 40 Gy (EQD2). The median prescribed dose in EQD2 was 40 Gy (18.95 Gy—74.75 Gy). The median prescribed dose in previous radiotherapy was 60 Gy (32 Gy—74.8 Gy) in 30 fractions (5–62 fractions). Toxicity was evaluated using the National Cancer Institute Common Toxicity Criteria version 4.0. The tumor stage was defined according to the Union for International Cancer Control (UICC) TNM Classification of Malignant Tumors version 7th. Informed consent was obtained from all subjects involved in the study at each institution. The study was conducted according to the guidelines of the Declaration of Helsinki and approved by the Institutional Review Board of the Kyoto Prefectural University of Medicine: ERB-C-1330-3.

### Statistical analysis

All statistical analyses were performed using Stat-view 5.0 statistical software (SAS Institute, Cary, NC, USA) and R-stat package^16^. Percentages were analyzed using the χ2 test, and values were compared using the Mann–Whitney *U* test^16^. The duration of survival was calculated from the first day of reirradiation. Actuarial survival curves were generated using the Kaplan–Meier method, and comparisons were made using the log-rank test^16^. For predisposing factor analysis for overall survival, we included age, sex, initial subsite, surgery, and lymph node involvement. Tumor size at recurrence (GTV: gross tumor volume), rT category, the interval between RT courses, chemotherapy, and prescribed dose in EQD2 used for reirradiation. We used GTV and prescribed a dose for local disease treatment regardless of lymph node involvement. Almost cases treated local and lymph node together with the same schedule, therefore GTV included both local and lymph node involvement. However, five cases treated local recurrence and lymph node involvement separately. We used GTV and schedule used for the treatment of local tumors in those cases. In addition, we used only GTV for multivariate analysis and not the rT category because there was a strong relationship between the rT category and GTV (correlation coefficient = 0.314, p = 0.00054). Variables were tested by multivariate analysis using a Cox proportional hazards model^16^. All analyses used the p < 0.05 level of significance unless otherwise indicated.

### Institutional review board statement

The study was conducted according to the guidelines of the Declaration of Helsinki and approved by the Institutional Review Board of the Kyoto Prefectural University of Medicine: ERB-C-1330-3.

### Informed consent

Informed consent was obtained from all subjects involved in the study.

## Results

### Patient and disease characteristics

The patient and disease characteristics are listed in Table 1. A total of 129 patients were eligible (95 men and 34 women) and the median age of patients was 63 years (range, 35–88 years). The most frequent primary sites were the nasopharynx (43.4%), oral cavity (24.8%), and oropharynx (18.6%). Chemotherapy was used on 3 patients. Lymph node involvement was found in 29 patients (21.2%). The median interval between initial radiotherapy and reirradiation was 19 months. Tumor volume was 23.7 cm3 in median (1 -339.6 cm^3^).

**Table 1:** Patient and treatment characteristics.

Factor	Group	Values
		(n = 129)
Age		65.00 [33.00, 88.00]
Gender	F	34 (26.4)
	M	95 (73.6)
Chemotherapy (%)	No	126 (97.7)
	Yes	3 ( 2.3)
Previous surgery (%)	No	79 (61.2)
	Yes	50 (38.8)
Primary site (%)	Hypopharynx	12 ( 9.3)
	Oral	32 (24.8)
	Larynx	5 ( 3.9)
	Nasopharynx	56 (43.4)
	Oropharynx	24 (18.6)
Histology	Squamous cell carcinoma	125 (97)
	Adenoid cystic carcinoma	1 (0.8)
	Adenocarcinoma	2 (1.5)
	Verrucous carcinoma	1 (0.8)
rT category	1	39 (30.2)
	2	29 (22.5)
	3	23 (17.8)
	4	36 (27.9)
	Not available	2 (1.5)
Lymph node involvement (%)	No	108 (78.8)
	Yes	29 (21.2)
GTV (cm^3^)		23.70 [0.91, 339.60]
Interval (months)		19.00 [3, 284.40]
Prescribed dose (Gy)		30.00 [15.00, 69.00]
fraction		5.00 [1.00, 30.00]
EQD2 (α/β = 10) (Gy)		40.00 [18.95, 74.75]
Previous prescribed dose (Gy)		60.00 [32.00, 74.80]
Previous fraction		30.00 [5.00, 62.00]
Follow-up times (months)		10.6 [0.10, 122]

*GTV* gross tumor volume.

**Figure 1:**
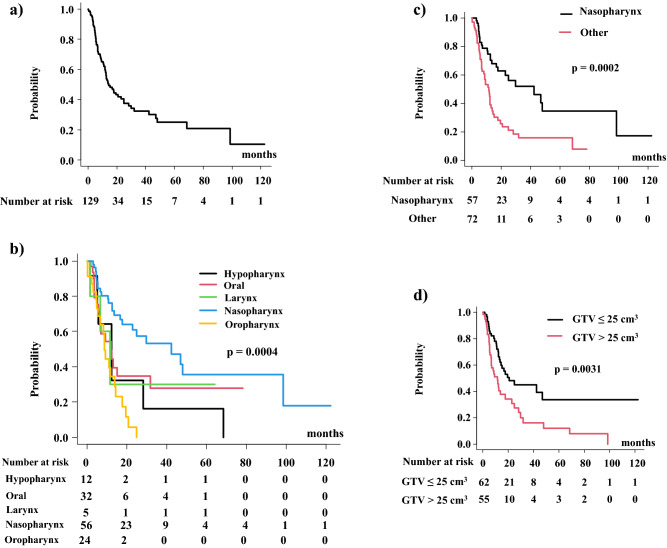
Overall survival rate (OS). (**a**) OS in the entire population. (**b**) OS according to primary sites. (**c**) OS according to nasopharyngeal cancer or not. (**d**) OS according to volumes of GTV.

The median follow-up duration for all patients was 10.6 (range, 0.1–122) months, the median overall survival time (MST) was 14.4 (95% confidence interval CI 12.0–24.8) months, and 1-year and 2-year overall survival rates were 59.0% (95% CI 49.1–67.5%) and 40.6% (95% CI 30.6–50.2%), respectively (Fig. 1a). For each primary site, the 2-year overall survival rates were 32.1% (95% CI 5.3–64.3%), 34.6% (95% CI 16.2–53.8%), 30% (95% CI 1.2–71.9%), 60.8% (95% CI 44.6–73.6%), and 5.7% (95% CI 0.3–22.8%) for the hypopharynx, oral cavity, larynx, nasopharynx, and oropharynx, respectively (p = 0.000407) (Fig. 1b).

Multivariate analysis revealed that the primary site (nasopharynx; hazard ratio = 2.96, p = 0.0024) and small GTV volume ≤ 25 cm3 (hazard ratio = 2.03, p = 0.013) were statistically significant favorable predictive factors for OS (Table 2). Age, gender, lymph node involvement, the interval between initial radiotherapy and reirradiation, previous surgery, chemotherapy, and prescribed dose in EQD2 were not statistically significant predisposing factors for overall survival.

**Table 2:** Multivariate analysis for overall survivai rate.

Factor	Strata	Hazard ratio	p value
Age	Sequential	1.01 (0.98–1.03)	0.54
Gender	M vs. F	0.85 (0.46–1.59)	0.62
Priamry site	Nasopharynx vs. other	2.96 (1.47–5.96)	**0.0024**
Lymph node invovement	Yes vs. no	1.42 (0.76–2.64)	0.28
Interval	≤ 12 months vs. > 12 months	0.99 (0.53–1.88)	0.98
Previous srugery	Yes vs. no	1.17 (0.66–2.07)	0.58
Chemotherapy	Yes vs. no	1.36 (0.30–6.16)	0.69
GTV	≤ 25 cm^3^ vs. > 25 cm^3^	2.03 (1.16–3.53)	**0.013**
EQD2	≤ 40 Gy vs. > 40 Gy	0.65 (0.38–1.11)	0.11

Significant values are in bold.

Patients with nasopharyngeal cancer showed a higher 2-years survival rate of 59.6% (95% CI 43.7–72.4%), than other primary sites of 23.5% (95% CI 12.8–36.0%, p = 0.0002, Fig. 1c). Patients with small GTV ≤ 25 cm3 showed a higher 48.1% (95% CI 33.5–61.4%) 2―year overall survival rate than those with large GTV > 25 cm^3^ of 30.8% (95% CI 17.2–45.5%, p = 0.00311, Fig. 1d). Patients with rT1 disease showed superior 2―year survival rates of 57.6% (95% CI 38.8–72.6%), compared with patients with rt2, rT3, and rT4 diseases with 2―year survival rates of 40.3% (21.6–58.2%), 32.1% (11.3–55.2%) and 23.2% (8.06–42.9%), respectively (Supplementary Fig. 1, p = 0.00627).

**Figure 2:**
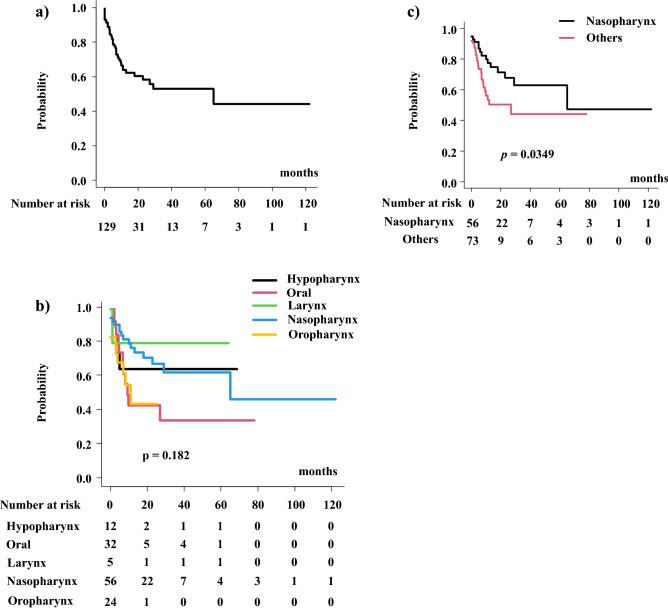
Local control rate. (**a**) Local control rate in the entire population. (**b**) Local control rate according to primary sites. (**c**) Local control rate between nasopharyngeal cancer and others.

Local failure was observed in 43 patients (33%) with an initial tumor progression. The local control rate was 63.8% (95% CI 53.2–72.6%) at 1-year and 58.2% (95% CI 46.7–68.1%) at 2 years (Fig. 2). For each primary site, the 2-year local control rate was 64.8% (95% CI 31.0–85.2%), 43.4% (95% CI 20.5–64.5%), 80% (20.4–96.9%), 68.0% (95% CI 51.0–80.0%), and 44.4% (95% CI 18.2–67.8%) for the hypopharynx, oral, laryngeal, nasopharynx, and oropharynx, respectively (p = 0.182) (Fig. 2b). In addition, there is a statistical difference between the nasopharynx and others. The 2-year local control rate was 68.0% (95% CI 51.0–80.0%) for nasopharyngeal cancer and 50% (95% CI 34.6–63.6%) for other primaries (Fig. 2c, p = 0.0293).

Nine patients showed distant metastases (3 skin, 3 liver, 2 supraclavicular lymph nodes, 3 lungs and/or mediastinal lymph node, 1 axillary lymph node, 1 peritonitis carcinomatosa, and 1 pleural effusion; three patients showed multiple metastases).

### Toxicity

A total of 24 patients (18.6%) presented with grade ≥ 3 toxicities, including seven with grade 5 (lethal toxicities, hemorrhage, and carotid blow-out syndrome), five with fistulas (3.9%), two with temporal lobe necrosis, one with bone necrosis/abscess, five with skin ulceration (with or without necrosis), one with visual disturbance, and one with long-term percutaneous endoscopic gastrostomy (Table 3).

**Table 3:** Toxicity.

Toxicity grade	Number of patients	(%)
(a) Frequency of maximal toxicity		
0	62	(48.1%)
1	9	(7.0%)
2	34	(26.4%)
3	13	(10.1%)
4	4	(3.1%)
5	7	(5.4%)
(b) Detail of toxicity grade ≥ 3 after reirradiation		
Meningitis	2	(1.6%)
Mucositis	2	(1.6%)
Visual disorder/blindness	6 (5 grade 4)	(4.7%)
Bone necrosis	2	(1.6%)
Sot tissue necrosis	1	(0.8%)
Trismus	1 (grade 5)	(0.8%)
Hemorrhaging	8	(6.2%)
Fistula	5	(3.9%)
Abscess	1	(0.8%)

Some patients had two or more toxicities.

There were 9 cases of hemorrhage (six with nasopharynx, two with oropharynx, and one with hypopharynx), of which 7 succumbed (one with grade 2 stopped without medication, one with grade 4 required interventional procedure, and seven with grade 5). Two patients with hemorrhage were treated using manual compression and/or interventional radiological procedures. All patients who experienced hemorrhages showed tumor involvement encasement into the carotid by > 180 degrees (360 degrees involvement in six cases). We examined the clinical characteristics that may predispose patients to hemorrhage; analysis suggested GTV as the only statistically significant predictor (Table 4).

**Table 4:** Background comparison between patients with or without bleeding.

Factor	Group	Hemorrhage (−)	Hemorrhage ( +)	p–value
		(n = 120)	(n = 9)	
Gender	Female	30 (25.0)	4 (44.4)	0.242
	Male	90 (75.0)	5 (55.6)	
Age	Sequential	67.00 [33.00, 88.00]	61.00 [44.00, 84.00]	0.149
Primary site	Hypopharynx	10 (8.3)	2 (22.2)	0.217
	Oral	32 (26.7)	0 (0.0)	
	Larynx	5 ( 4.2)	0 (0.0)	
	Nasopharynx	51 (42.5)	5 (55.6)	
	Oropharynx	22 (18.3)	2 (22.2)	
GTV	≤ 25 cm3	61 (56.5)	1 (11.1)	**0.012**
	> 25 cm3	47 (43.5)	8 (88.9)	
Lymph node involvement	No	95 (79.1)	6 (66.7)	0.407
	Yes	26 (21.6)	3 (33.3)	
Local control	Yes	83 (69.2)	3 (33.3)	0.059
	No	37 (30.8)	6 (66.7)	
Chemothrerapy	No	117 (97.5)	9 (100.0)	1
	Yes	3 ( 2.5)	0 (0.0)	
Surgery	No	72 (60.0)	7 (77.8)	0.481
	Yes	48 (40.0)	2 (22.2)	
Interval	(month)	19.30 [0.30, 284.40]	11.50 [1.50, 92.70]	0.302
RT dose EQD2 (a.b.3)	(Gy)	53.39 [20.80, 124.80]	43.63 [24.31, 108.00]	0.468

Significant values are in bold.

## Discussion

Here, we present the reirradiation outcomes for local recurrence of oral, pharyngeal, and laryngeal cancers with or without regional recurrence. To the best of our knowledge, this is one of the largest cohorts of patients who received reirradiation focused on local recurrence of oral, pharyngeal, and laryngeal cancers.

The management of recurrence in the head and neck remains challenging because of the high doses of radiation therapy required during the initial curative treatment of the primary disease^1–3^. Salvage surgery is the treatment of choice; however, only a small number of patients can undergo salvage surgery and receive systemic therapy, leading to overall poor outcomes^4,5^. Initial prospective multi-institutional trials for reirradiation used two―dimensional or three―dimensional radiotherapy. RTOG 9610 used twice a day for radiation up to 60 Gy in 40 fractions with concurrent fluorouracil and hydroxyurea weekly for unresectable recurrent squamous cell carcinoma of the head and neck^17^. The 2-year survival rate was 15% (MST 8.5 months) with significant acute grade 4 and 5 toxicity (18 and 7.6%). They found that the interval between initial treatment and reirradiation was an important predisposing factor (interval less than 12 months vs. more than equal 12 months; MST 12.1 months vs. 5.8 months). RTOG 9911 installed a new chemotherapy regimen (combined with paclitaxel and cisplatin) using every other week split-dosing of radiotherapy for reducing toxicity, which resulted in an improved MST of 12.1 months, and 2―year overall survival was 25.9%^18^. However, toxicity remained severe; acute grade 3 or more and grade 5 were 78% and 7.6%. Strojan et al. reviewed reirradiation to the head and neck lesions in a heterogeneous population majorly treated by two―dimensional or three―dimensional radiotherapy and reported that the 2-year overall survival rate was generally 10–30%^19^. Late toxicity grades 3–4 were common in 40%, and grade 5 due to carotid rupture, hemorrhage, sepsis, etc. were found in approximately 10%^19^.

Advanced radiotherapy techniques, such as IMRT and SBRT, have enabled the delivery of a higher radiation dose to the tumor without sparing the surrounding organ at risk; therefore, reirradiation is a potentially curative therapy^6–12^. Lee et al. conducted a meta-analysis of IMRT/ SBRT and found a 2-year survival rate of 30—46%; which seems superior to previous outcomes obtained with two―dimensional or three―dimensional radiotherapy^20,21^. However, reirradiation is challenging because of the complexity of the tolerance of various normal tissues after initial radiotherapy. Previously reported prognostic factors after reirradiation include primary site (i.e. nasopharyngeal primary site vs. other sites), histology (i.e. squamous cell carcinoma [SCC] or not), the interval between treatment, tumor or treatment volume, surgical procedure, and prescribed dose^22–24^. Lee et al. reported frequency of toxicities of grade ≥ 3 was 9.6—26%^20,21^ which may be lower than the previous two- or three―dimensional radiotherapy era, however also retains a substantial influence on patients’ quality of life.

Ward et al. performed a recursive partitioning analysis (RPA) in patients treated with IMRT reirradiation using the following selection criteria: (i) patients with an inter-treatment interval > 2 years and classified into Class I (2y-OS, 61.9%); (ii) patients with an inter-treatment interval > 2 years, but not resected, or those with inter-treatment interval ≤ 2 years with no organ dysfunction (feeding tube or tracheostomy dependence), classified into Class II (2y-OS, 40%); and (iii) patients with organ dysfunction classified into Class III (2-y OS, 16.8%). In the present study, almost the entire cohort may belong to Class II and have 2y-OS 40%^25^. Vargo et al. adapted the classification for both IMRT and SBRT; analysis by RPA class showed similar OS between the IMRT and SBRT groups for Class III patients^24^. In other classes, IMRT was associated with improved OS (especially for rT3–4 and prescribed dose ≥ 66 Gy), except when ≥ 35 Gy was administered with SBRT to small tumor volumes ≤ 25 cm^3^^18–25^. Additionally, primary sites, such as the oral cavity, oropharynx, larynx, and hypopharynx showed poorer prognoses than other sites, not only for the nasopharynx, but also for the nasal sinus, paranasal sinus, parotid, orbit, and thyroid in several studies^22–28^. Therefore, a major part of our study cohort may belong to the poor prognosis category in the entire head and neck reirradiation series.

The delayed severe adverse events are a major obstacle to reirradiation. Grade 3–4 was common in 40%, and grade 5 toxicity, including hemorrhage (CBOS) and sepsis, was found in approximately 10% of the patients^18^. For example, CBOS is one of the most devastating toxicities that occur following reirradiation^7,8,12–15,28^. Chloe et al. reported that 15 out of 33 treatment-related deaths (40%) were related to CBOS in 166 patients (overall rate = 9%)^29^. The present study also indicated CBOS in 5.4% of 129 patients with oral, laryngeal, and pharyngeal carcinoma, consistent with the results of previous studies. We have previously explored CBOS; the analyses suggested CBOS in 8.4% of 381 patients with head and neck carcinoma treated with 484 reirradiation sessions at seven Japanese CyberKnife institutions, of which 69% were fatal^13,14^. Additionally, the presence of ulceration associated with carotid invasion > 180° is an important risk factor for CBOS^13,14^. In recent SBRT-based approaches, CBOS has also been correlated with tumors > 26 cc, carotid invasion ≥ 180°, and doses > 34 Gy in five fractions^22,30^. The findings of the present study concur with those of previous studies, as all nine tumors that caused hemorrhage involved the carotid > 180 degrees and larger GTV than non-hemorrhagic tumors. The Hypofractionated Treatment Effects in the Clinic (HyTEC) group found that a maximum dose to the major vessels of 20–30 Gy in 5 fractions was generally low risk, but risk increased quickly > 30 Gy to a tolerance dose of 45.7 Gy^15,22,30^. The other factors that reduced the rate of CBOS were alternate day treatment rather than daily treatment, not treating patients with skin invasion, necrosis, or infection, and avoiding postoperative treatment^15^. We concur with these considerations. We reported the initial results in 2013^13,14^; after which, we focused on the risk factors mentioned above and developed strict patient selection criteria with alternate day treatment. No cases of hemorrhage have been reported since then, although the number of patients has been small.

Combination therapy with systemic therapy should be considered to enhance efficacy, although no randomized controlled trial compares the combination treatment with radiotherapy alone^31–34^. Awan et al. reported the results of a phase II clinical trial using a combination of concurrent cisplatin and cetuximab with IMRT ≥ 60 Gy reirradiation (2-year OS: 50.4% for patients with preceding surgery versus 33.3% for those without)^33^. Furthermore, radiotherapy can induce local and abscopal antitumor immune responses^28^. SBRT with hypofractionation leads to greater immune stimulation than conventional fractionation^28^. With the advent of immune checkpoint inhibitor therapy, the RTOG 3507 study, started in 2018, randomized patients to receive SBRT at a dose of 40 Gy in 5 fractions over 2 weeks with or without concurrent and adjuvant pembrolizumab for up to 2 years^35^.

This study has some limitations. First, the retrospective nature, and small cohort size, especially for subgroups (i.e. laryngeal cancer, heterogeneous treatment schedules, and limited follow-up time) limit its application. A prospective trial with a larger number of patients and a longer follow-up period should be performed to confirm the findings. Second, we were unable to analyze the details of previous chemotherapy and/or surgery due to the large heterogeneity in reporting practices between the institutions. Third, a detailed dose―volume analysis for individual cases could not be performed due to a lack of information. Composite dose―volume analysis is important for predicting tumor control and toxicity, for both hemorrhage and other toxicities, such as mucositis, necrosis, and fistula. At last, we treated patients with advanced technique (SBRT and/or IMRT) which is not available in many institutions across the globe, especially in Low to middle―income Group countries, which bear the burden of Head Neck cancer—generalizing these results may not apply to the global setting at large.

## Conclusions

Reirradiation is a feasible treatment option for the small local recurrence of oral, pharyngeal, and laryngeal cancers, with a strict eligibility assessment for large tumors with carotid encasement.

## Supplementary Information

Supplementary Figure S1.

## Data Availability

The datasets used and/or analysed during the current study are available from the corresponding author upon reasonable request.
