# Supplementary material for: Reirradiation for local recurrence of oral, pharyngeal, and laryngeal cancers: a multi-institutional study
Source: Sci Rep. 2023 Feb 21;13:3062. doi: 10.1038/s41598-023-29459-2 (PMC9944926; doi:10.1038/s41598-023-29459-2)
Supplement: Supplementary file 1 — Supplementary Figure S1. [file 41598_2023_29459_MOESM1_ESM.pptx]

## Slide 1
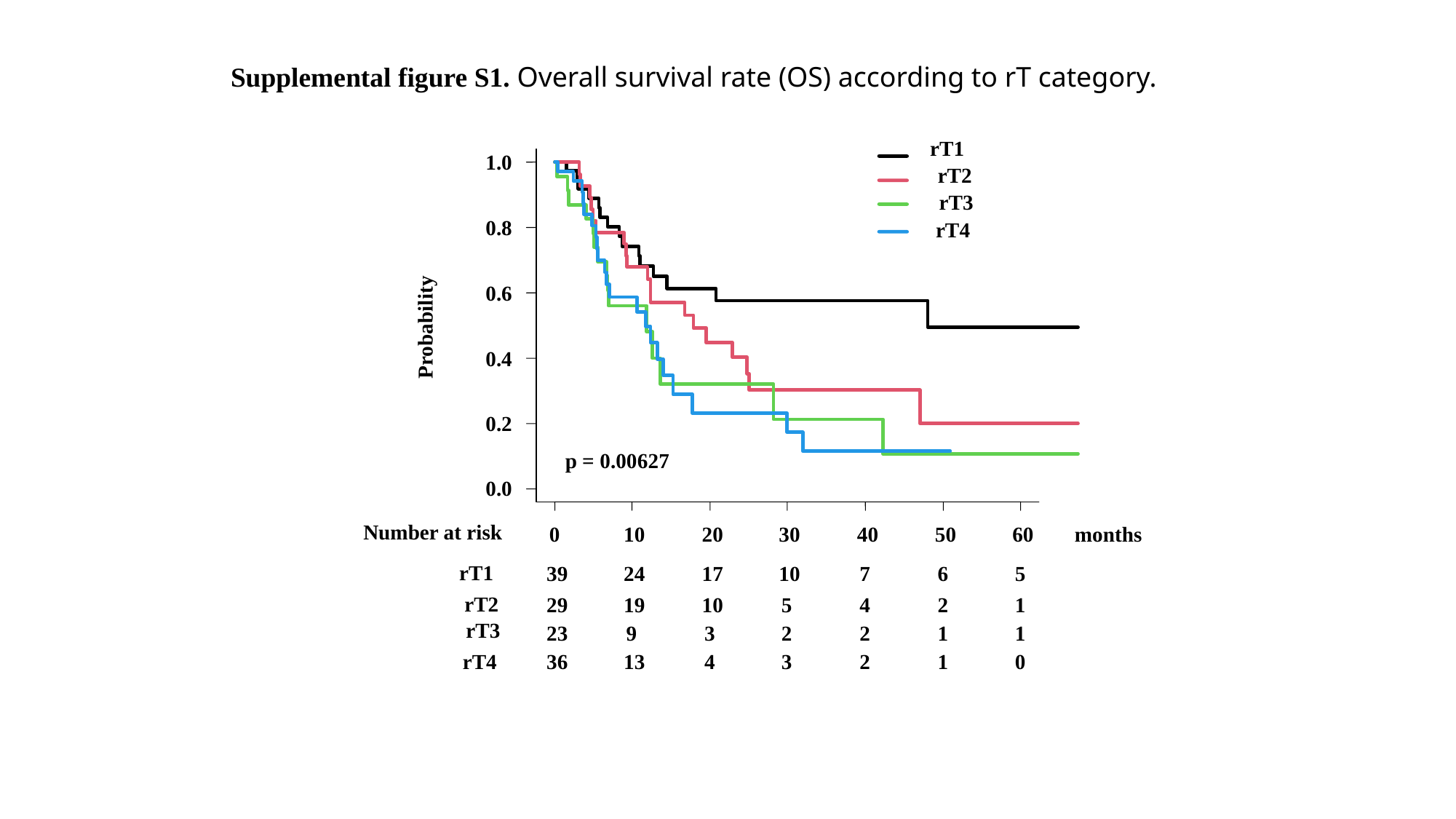

Supplemental figure S1. Overall survival rate (OS) according to rT category.
rT1
1.0
rT2
rT3
0.8
rT4
0.6
Probability
0.4
0.2
p = 0.00627
0.0
Number at risk
0
10
20
30
40
50
60
months
rT1
39
24
17
10
7
6
5
rT2
29
19
10
5
4
2
1
rT3
23
9
3
2
2
1
1
36
13
4
3
2
1
0
rT4
